# Why more successful? An analysis of participants’ self-monitoring data in an online weight loss intervention

**DOI:** 10.1186/s12889-024-17848-9

**Published:** 2024-01-29

**Authors:** Hai-Bo Tang, Nurul Iman Binti Abdul Jalil, Chee-Seng Tan, Ling He, Shu-Juan Zhang

**Affiliations:** 1 Faculty of Education, Yibin University, Yibin, 644000 China; 2 Department of Psychology and Counselling, Universiti Tunku Abdul Rahman, Kampar, 31900 Malaysia; 3 School of Psychology, College of Liberal Arts Wenzhou-Kean University, Wenzhou, Zhejiang province 325060 China; 4 Sichuan Tianfu New District No. 3 Middle School, Chengdu, 610213 China

**Keywords:** Online intervention, Content analysis, Self-monitoring, Weight loss, Group counseling

## Abstract

**Background:**

Self-monitoring is crucial for behavioral weight loss. However, few studies have examined the role of self-monitoring using mixed methods, which may hinder our understanding of its impact.

**Methods:**

This study examined self-monitoring data from 61 Chinese adults who participated in a 5-week online group intervention for weight loss. Participants reported their baseline Body Mass Index (BMI), weight loss motivation, and engaged in both daily quantitative self-monitoring (e.g., caloric intake, mood, sedentary behavior, etc.) and qualitative self-monitoring (e.g., daily log that summarizes the progress of weight loss). The timeliness of participants’ daily self-monitoring data filling was assessed using a scoring rule. One-way repeated measurement ANOVA was employed to analyze the dynamics of each self-monitoring indicator. Correlation and regression analyses were used to reveal the relationship between baseline data, self-monitoring indicators, and weight change. Content analysis was utilized to analyze participants’ qualitative self-monitoring data. Participants were categorized into three groups based on their weight loss outcomes, and a chi-square test was used to compare the frequency distribution between these groups.

**Results:**

After the intervention, participants achieved an average weight loss of 2.52 kg (*SD* = 1.36) and 3.99% (*SD* = 1.96%) of their initial weight. Daily caloric intake, weight loss satisfaction, frequency of daily log, and the speed of weight loss showed a downward trend, but daily sedentary time gradually increased. Moreover, regression analysis showed that baseline BMI, weight loss motivation, and timeliness of daily filling predicted final weight loss. Qualitative self-monitoring data analysis revealed four categories and nineteen subcategories. A significant difference in the frequency of qualitative data was observed, with the excellent group reporting a greater number of daily logs than expected in all categories and most subcategories, and the moderate and poor groups reporting less than expected in all categories and most subcategories.

**Conclusion:**

The self-monitoring data in short-term online group intervention exhibited fluctuations. Participants with higher baseline BMI, higher levels of weight loss motivation, and timely self-monitoring achieved more weight loss. Participants who achieved greater weight loss reported a higher quantity of qualitative self-monitoring data. Practitioners should focus on enhancing dieters’ weight loss motivation and promote adherence to self-monitoring practices.

## Introduction

Obesity has become a worldwide public health problem. According to the World Health Organization [1], more than 1.9 billion adults over the age of 18 were overweight in 2016, and more than 650 million of them were obese. In China, the proportion of overweight and obese people is alarming. Recent data show that more than half of the people aged 18 and above are overweight (34.3%) or obese (16.4%) [2]. Obesity is a serious threat to people’s health, increasing the risk of diseases (e.g., diabetes, cardiovascular disease, and cancer) [3, 4] and impairing individuals’ mental health [5]. During the COVID-19 pandemic, obesity also exacerbated the condition of patients after infection with the virus, leading to worse outcomes [6].

Self-monitoring is a systematic process aimed at enhancing self-awareness, promoting desired behaviors, and reducing unwanted behaviors through the observation, measurement, and logging of specific target behaviors [7, 8]. Within the context of weight loss interventions, participants engage in regular self-monitoring by documenting their dietary intake, exercise routines, and changes in body weight [9, 10]. Studies have demonstrated that self-monitoring can serve as an effective predictor of weight loss [8, 11, 12]. For instance, overweight or obese adults who consistently monitor their diet and body weight have reported greater weight loss compared to those who do not engage in self-monitoring practices [12]. Interestingly, the continuity of self-monitoring appears to be more influential than the specific method employed for monitoring. Research has indicated that the method of self-monitoring, such as using a paper diary or an Internet-based tool [13], and the level of detail recorded during self-monitoring [14] do not affect weight loss outcomes. Furthermore, the order in which self-monitoring is conducted, whether recording both diet and weight simultaneously or recording weight first and subsequently adding the diet, has been found to have no impact on weight loss outcomes [15]. Of greater importance is the frequency of self-monitoring, which has been shown to positively correlate with weight loss [16].

Some analyses of self-monitoring behaviors among successful and unsuccessful dieters have revealed valuable findings. Firstly, successful dieters tend to utilize self-monitoring strategies more frequently. For example, a retrospective analysis of a database, which tracked participants for 10 years, revealed that decreased self-weighing, as well as reduced leisure-time physical activity and dietary restrictions, were positively associated with greater weight regain [17]. Additionally, qualitative studies conducted on both adolescents [18] and adults [10] have reported that consistent tracking of diet, physical activity, and weight led to more successful weight loss compared to those who did not engage in such monitoring. Secondly, successful dieters are better prepared to navigate high-risk periods during their weight loss journey. Weekends and public holidays, known to pose challenges for individuals seeking weight loss [19, 20], are particularly vulnerable times. Successful dieters demonstrate the ability to maintain their regular dietary habits and engage in compensatory behaviors, such as exercise, during these high-risk periods [20, 21].

Although research has been conducted on quantitative and qualitative self-monitoring behaviors of dieters, few studies have employed a mixed-methods approach to investigate this issue, which may lead to limitations in the understanding of dieters’ self-monitoring behaviors. A mixed methods research can reveal the relationship between different self-monitoring indicators and weight loss, and also keep the research perspective open to develop a more complete insight into self-monitoring.

To address these gaps, this study focused on two key aspects. Firstly, using quantitative data, we aimed to uncover the changes in participants’ self-monitoring behavior throughout the intervention period and examine the relationship between self-monitoring behavior and the final weight loss. Secondly, through qualitative data analysis, we aimed to explore how participants summarized their daily weight loss process and understand how these summaries were related to their overall weight loss outcomes. By integrating these quantitative and qualitative data aspects, our study aimed to develop a more comprehensive understanding of self-monitoring during short-term online group intervention.

## Method

### Participants

All participants were recruited from China and through social networking sites (e.g., WeChat) with a convenient sampling. Researchers disseminated recruitment advertisements to potential participants through WeChat groups and Moments. Additionally, recruited participants were encouraged to share the recruitment information on their own WeChat Moments to broaden outreach. Given the prevalence of mild weight loss aspirations and behaviors among individuals with normal weight in China [22], this study extended its recruitment to include individuals within the normal weight range who expressed a willingness to lose weight. Due to the higher body fat percentage observed among Asian individuals with the same BMI [3], we have adopted the Chinese classification standard for obesity, which categorizes a BMI of 24 or above as overweight and a BMI of 28 or above as obesity [2]. The eligibility criteria for recruitment included having the intention to lose weight, proficiency in using smartphones, absence of any mental illness or conditions unsuitable for weight loss, and no participation in other weight loss interventions in the past six months. Participants were given a brief online interview to ensure a genuine desire to lose weight and to meet the recruitment criteria. A total of 64 participants participated in the online intervention, and data from 61 participants (including 3 males) were included in the analysis. The exclusion criteria led to the exclusion of 2 participants who filled in less than 1/3 of the required number of days to be filled and 1 participant who reported many outliers. Participants’ age ranged from 18 to 51 years old, with a mean age of 30.0 (*SD* = 8.15), and a mean BMI of 24.14 kg/m^2^ (*SD* = 3.13). Among the participants, 35 individuals had a normal weight, with a mean BMI of 22.16 kg/m^2^ (*SD* = 1.11), while 26 participants were classified as overweight or obese (19 were overweight and 7 were obese), with a mean BMI of 26.81 kg/m^2^ (*SD* = 2.99). Furthermore, to compare the differences in participants’ qualitative self-monitoring data, we categorized them into the Excellent group (greater than or equal to 5%, *n* = 20), Medium group (greater than or equal to 3%, *n* = 22), and Poor group (less than 3%, *n* = 19) based on the percentage of weight loss. There were no significant differences in the age and baseline BMI among the three groups of participants except for weight loss motivation (Table 1).

**Table 1:** Participants’ demographic information

	Total (*n* = 61)	Poor group (*n* = 19)	Medium group (*n* = 22)	Excellent group (*n* = 20)	*p*
Age	30.00 ± 8.15	33.16 ± 7.96	27.82 ± 8.82	29.40 ± 6.91	0.102
BMI	24.14 ± 3.13	23.51 ± 1.95	25.13 ± 4.32	23.64 ± 2.23	0.178
WLM	3.59 ± 0.96	3.26 ± 0.81	3.46 ± 0.86	4.05 ± 1.05	0.023

Note. BMI = Body mass index; WLM = Weight loss motivation; WLM_poor group_ < WLM_excellent group_

### Instruments and measurement

#### Online group counseling intervention

All participants were randomly assigned to six online groups, each comprising 8–12 participants. These groups were closed, and no new members were admitted during the intervention period. The online intervention consisted of five sessions, each lasting 90 min, conducted over five consecutive weeks (one session per week).

The first session, titled “Serendipitous Encounters,” aimed to foster mutual familiarity, establish group rules, and ignite motivation for weight loss. The second session, focused on “Healthy Eating,” aimed to assist participants in cultivating healthy lifestyle habits and acquiring dietary knowledge conducive to weight reduction. The core concepts conveyed by researchers to participants include the freedom to choose foods with a restriction on overall calorie intake, the practice of mindful eating, and the pursuit of nutritional balance. In the third session, titled “Bidding Farewell to Impulsive Eating,” typical impulsive eating behaviors in participants’ lives were addressed, along with discussions on coping strategies. The fourth session, themed “Emotional Management,” sought to enhance participants’ coping abilities with negative emotions, thereby reducing emotional eating. The fifth and final session, titled “Forever on the Journey,” aimed to help participants embrace the concept of lifelong weight management, cultivate a healthy lifestyle, embrace self-acceptance, and maintain weight within a normal range.

For the intervention sessions, the Tencent Meetings app, a reputable Chinese internet video conferencing platform, was utilized. The intervention groups were led by a certified counselor with over 10 years of experience in psychological counseling. The intervention took place between June and September 2022. Outside of the intervention sessions, participants had the opportunity to communicate and remind each other to complete their self-monitoring tasks in a WeChat group.

### Quantitative self-monitoring data

Participants were instructed to engage in self-monitoring throughout the weight loss program. The quantitative self-monitoring encompassed various aspects, including tracking daily caloric intake, daily physical activity expenditure, daily sedentary time, daily mood, and daily weight loss satisfaction.

Consistent with previous research [23, 24], participants utilized smartphones to track their daily caloric intake and daily physical activity expenditure. Using the Bohee Health application, a leading weight loss app in China, participants logged their daily physical activity types and durations, as well as the types and weights of consumed food. The app automatically calculated the daily caloric intake and physical activity expenditure based on the provided data. To improve the precision of participants’ assessments of food weight, researchers provided the weights of typical food portions and encouraged participants to use food scales for measuring the weights of the food they regularly consumed. Additionally, participants self-reported their daily sedentary hours, which were also recorded for analysis.

The single-item assessment has demonstrated satisfactory reliability in previous research on emotions and self-esteem [25, 26]. To minimize participants’ burden, this study employs a self-developed single-item measure to assess overall daily emotions and daily satisfaction with their weight loss progress. Specifically, participants’ daily mood was evaluated using a question: “How are you feeling today?” Participants rated their overall mood on a scale ranging from 1 (very bad) to 10 (very good). In addition, participants’ daily weight loss satisfaction was assessed with the question: “Are you satisfied with your weight loss performance today?” Participants indicated their level of satisfaction on a scale from 1 (very dissatisfied) to 10 (very satisfied).

To track participants’ weight changes, baseline weight was measured on the morning of the first session. Subsequently, participants were instructed to weight themselves at the same time each week, wearing light clothing and being barefoot. The final weight change was calculated by subtracting the weight measured in the sixth week (i.e., one week after the fifth online session) from the baseline weight.

### Qualitative self-monitoring data

Participants’ qualitative self-monitoring data were obtained through an open-ended question. While participants were encouraged, but not obligated, to log a concise summary of their daily weight loss performance in a few short sentences (hereinafter called daily log), they had the flexibility to document any behaviors, thoughts, or feelings related to their weight loss journey for that day.

### Other data

During enrolment, participants provided their age and height through self-reporting. Their weight data from the first session was combined with height to calculate their baseline BMI. Participants’ weight loss motivation was assessed using a self-report question during the first session: “Objectively, how would you assess your motivation level for weight loss?” Participants rated their weight loss motivation on a scale from 1 (very low) to 5 (very high).

All self-monitoring data were required to be filled in the “Jielong Guanjia” application on WeChat by 10:30 p.m. each day. The timeliness of daily data filling was evaluated using a scoring system. The rule was as follows: a score of “3” for filling in the data within the required time, a score of “2” for filling in the data late on the same day, a score of “1” for filling in the data on the next day, and a score of “0” for not filling in the data. The participant’s final score was calculated by multiplying the ratio of the actual score to the theoretical total score by 100. A higher score indicates more timeliness in filling in the data.

Additionally, to gather more valuable information, the frequency of participants’ interactions with group members was also collected after the fifth week. The frequency of interaction with group members (hereinafter referred to as group interaction) was calculated individually for each participant. Each message posted by a participant, including separate emojis, was counted as one interaction.

### Data analysis

Both quantitative and qualitative data were collected in the present study. The mean of the participants’ daily self-monitoring (quantitative) data for each week was calculated and used for further statistical analysis. The quantitative data were analyzed using JASP (JASP Team 2022; Version 0.16.3). To reveal the changes in participants’ self-monitoring data, one-way repeated measures analysis of variance (ANOVA) was used to examine whether there were differences in the weekly mean values of the self-monitoring data (e.g., daily caloric intake) during the weight-loss period. Next, Pearson correlation analysis was performed to examine the relationships between self-monitoring quantitative data, background information (e.g., BMI, weight loss motivation), and weight loss. Lastly, multiple linear stepwise regression was employed to identify significant predictors of weight loss.

Two coders conducted content analysis on the qualitative data of participants’ daily log text, following a five-step process recommended in previous research [27, 28]. The content analysis steps are as follows: In the first step, the entire text content was read immersively to understand the overview and intrinsic connections of the text data. The second step was to split the text information with multiple meanings to form single-meaning units. For instance, a participant summarized in her daily log, “These two days, my diet has been irregular, and I haven’t exercised. My weight is decreasing in an unhealthy manner.” This log is segmented into three meaning units: “Irregular diet these two days,” “No exercise,” and “Unhealthy weight decrease.” The third step was open coding. The meaning units are openly encoded, with a core concept representing its meaning while maintaining as much as possible the original appearance. Meaning units with similar meanings were merged. For example, the " irregular diet these two days " was coded as “irregular diet.” The meaning units “ran 2 kilometers,” “started running,” and “ran 5 laps at the stadium” were coded as “Running.” In the fourth step, subcategories were formed. Similar codes are formed into subcategories. As an example, activities such as “walking,” “running,” “aerobics,” “swimming,” and “hiking” were categorized under “Type of exercise”. The last step was to form categories. Similar subcategories were further organized to form categories. Adjustments were allowed throughout the analysis process to ensure reasonableness. To ensure the quality of the analysis, the first coder (the first author) performed analysis and coding, and the second coder (the fourth author) checked after the preliminary analysis. In cases of disagreement, the two coders engaged in discussions to reach a consensus. If a consensus could not be reached, an expert was consulted to review and make a final decision.

To reveal differences in qualitative self-monitoring data between successful and unsuccessful dieters, participants were divided into 3 groups based on their weight loss performance. Considering that 5% weight loss is usually considered clinically significant [29, 30], and 3% weight loss is also considered successful [31], we divided participants into 3 groups: the poor group (weight loss < 3%, *n* = 19), the moderate group (3% ≤ weight loss < 5%, *n* = 22), and the excellent group (weigh loss ≥ 5%, *n* = 20). There were no significant differences in the baseline BMI among the three groups of participants (*F* = 1.779, *p* =.178, η²**=** 0.058). The chi-square test was employed to assess whether the frequency of meaning unit distributions reported by the three groups of participants in each category and subcategory followed the same probability distribution.

## Results

### Changes in self-monitoring quantitative data

**Fig. 1:**
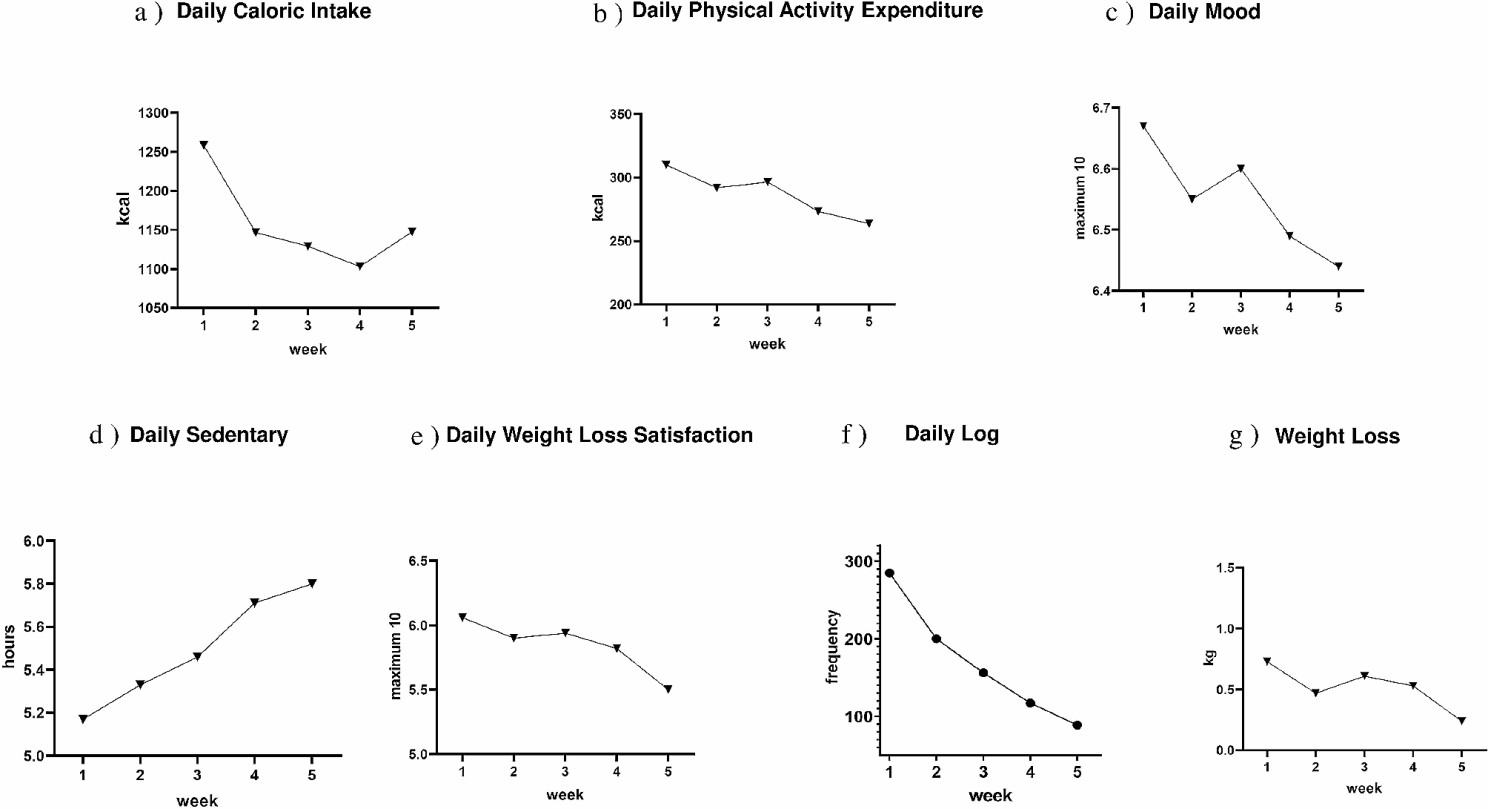
Changes in participants’ self-monitoring data

After the intervention, participants lost an average of 2.52 kg (*SD* = 1.36) and 3.99% (*SD* = 1.96%) compared to their baseline weight. The results of the One-Way repeated measures ANOVA revealed significant differences in some self-monitoring quantitative measures over the 5-week weight loss period (Fig. 1). Specifically, there was a gradual decrease in participants’ caloric intake (Fig. 1a), *F* (2.246, 128.014) = 4.066, *p* =.016, ω² = 0.016. The average daily caloric intake in week 1 was significantly higher than the intake in week 3 (*t* = 3.100, *p* =.020) and week 4 (*t* = 3.720, *p* =.003), respectively. On the other hand, participants’ daily physical activity expenditure also showed a slight decrease over the 5-week period (Fig. 1b), but the difference was not significant, *F* (2.823, 160.917) = 2.011, *p* =.118, ω²**=**. 005. Similarly, participants’ daily moods remained stable over the 5 weeks (Fig. 1c) and significant differences were not found among the weekly mean scores, *F* (3.512, 200.183) = 0.931, *p* =.438, ω² = 0.000. Surprisingly, participants’ daily sedentary hours showed an unexpected increasing trend over the 5 weeks (Fig. 1d), *F* (3.196, 182.162) = 4.308, *p* =.005, ω² = 0.013. The averaged sedentary hours in week 4 (*t* = -3.036, *p* =.024) and week 5 (*t* = -3.516, *p* =.005) were significantly higher than in week 1.

Participants also exhibited a downward trend in their overall satisfaction with their daily weight loss (Fig. 1e), *F* (3.612, 205.902) = 4.370, *p* =.003, ω² = 0.013. Participants reported a higher satisfaction in week 1 (*t* = 3.905, *p* =.001), week 2 (*t* = 2.826, *p* =.041), and week 3 (*t* = 3.117, *p* =.019) than in week 5. Notably, there was a gradual decrease in the number of daily weight loss summaries reported by participants (Fig. 1f), *F* (2.623, 157.4) = 15.777, *p* <.001, ω² = 0.084. Specifically, the frequency reported in week 1 was significantly higher than in week 2 (*t* = 3.102, *p* =.013), week 3 (*t* = 4.708, *p* <.001), week 4 (*t* = 6.131, *p* <.001), and week 5 (*t* = 7.153, *p* <.001). Additionally, participants reported more frequently in week 2 than in week 4 (*t* = 3.029, *p* =.014) and week 5 (*t* = 4.051, *p* <.001). Finally, weekly weight loss showed a similar downward trend (Fig. 1g), *F* (4, 212) = 3.525, *p* =.008, ω² = 0.038, with significantly more weight loss in week 1 than in week 5 (*t* = 3.579, *p* =.004).

### Predictors of weight loss

To reveal the relationship between participants’ weight loss and potential predictors, a Pearson correlation analysis was conducted. As shown in Table 2, weight loss motivation, timeliness of daily filling, and daily log frequency were found to have a weak to moderate, significant positive correlation with participants’ weight loss (%), respectively. In addition, participants’ baseline BMI, weight loss motivation, timeliness of daily filling, and daily log were positively correlated with weight loss (kg).

**Table 2:** Pearson correlation between participants’ weight loss, self-monitoring and background data

	Age	BMI	WLM	DCI	DPAE	DM	DS	DWLS	DL	TDF	GI
WL (%)	-0.119	0.069	0.33^**^	-0.094	0.155	0.161	-0.173	0.226	0.29^*^	0.318^*^	0.168
WL (kg)	-0.118	0.38^**^	0.398^**^	0.003	0.232	0.119	-0.081	0.216	0.288^**^	0.328^*^	0.233

Note. *N* = 61. BMI = Body mass index; WLM = Weight loss motivation; DCI = Daily caloric intake (kcal); DPAC = Daily physical activity expenditure (kcal); DM = Daily mood; DS = Daily sedentary; DWLS = Daily weight loss satisfaction; DL = Daily log (frequency); TDF = Timeliness of daily filling; GI = Group interaction (frequency); WL (%) = Weight Loss (%); WL (kg) = Weight loss (kg)

^*^p<05; ^**^p<.01; ^***^p<.001

Multiple linear regression analyses were performed to identify the predictors of weight loss percentage and absolute weight loss, respectively (Table 3). Only potential predictor variables that showed a significant correlation with the dependent variable were selected as independent variables in the regression analysis.

**Table 3:** Multiple stepwise regression analysis of final weight loss

	Model 1: Weight Loss (%)			Model 2: Weight loss (kg)		
Predictors	β	t	*p*	β	t	*p*
BMI	-	-	-	0.269	2.367	0.021
WLM	0.311	2.636	0.011	0.313	2.773	0.007
TDF	0.297	2.519	0.015	0.274	2.482	0.016
*R*²(adjusted *R*²)	0.197 (0.169)			0.317 (0.281)		
Model *F*	7. 117 (*p* =.002)			8.816 ( *p*<.001 )		

Note. BMI = Body mass index; WLM = Weight loss motivation; TDF = Timeliness of daily filling; In both Model 1 and Model 2, Weight loss summary (frequency) was excluded from the final model in the stepwise regression analysis

In the first regression model, the outcome variable was the weight loss (%). Weight loss motivation, daily log, and timeliness of daily filling as independent variables. The independent variables had low correlations with each other (correlation coefficients ranging from 0.066 to 0.308). The Variance Inflation Factor (VIF) for independent variables ranged from 1.021 to 1.124 (VIF < 10), and the tolerance was range from 0.890 to 0.979 (tolerance > 0.1), implying that there was no obvious colinearity between the independent variables [32]. The QQ plots of the residuals showed that the standardized residuals are distributed along the diagonal, which suggests that the residuals are normally distributed. In addition, the Durbin-Watson value was 2.456, implying that there was no autocorrelation in the adjacent error terms. Analysis results showed that the daily log was excluded from the regression model (stepwise method). Lastly, weight loss motivation and timeliness of daily filling effectively predicted weight loss (%) and explained 19.7% of the total variance.

In the second regression model, the weight loss (kg) was regressed on baseline BMI, weight loss motivation, timeliness of daily filling, and daily log. Similarly, the regression analysis also met the prerequisites, as the independent variables exhibited low correlation with each other (correlation coefficients ranging from 0.066 to 0.308). The VIF values ranged from 1.034 to 1.167, and the tolerance values ranged from 0.857 to 0.928, indicating no obvious colinearity. The Durbin-Watson value was 2.320, indicating no significant autocorrelation. Regression analysis revealed that baseline BMI, weight loss motivation, and timeliness of daily filling positively predicted weight loss (kg), explaining 31.7% of the variance in weight loss.

### Participants’ daily log

After conducting a content analysis on participants’ daily log, a total of 847 meaningful units (93.18% of the raw data’s total frequency) were included in the final categories. The textual information provided by participants during the weight loss process fell into four main categories: *weight loss awareness*, *eating behavior*, *physical activity*, and *perception of change* (Table 4). The category of weight loss awareness encompassed participants’ perception of weight loss developed throughout the weight loss process and included six subcategories: risk situations, self-motivation, disadvantages, maintaining awareness, follow-up plan, and patience. Eating behavior referred to participants’ descriptions of their daily eating habits. It consisted of six subcategories: restrictive eating, overeating, inhibiting failure, irregular eating, mindful eating, and intuitive eating. Physical activity captured participants recorded physical activities and comprised three subcategories: exercise amount, type of exercise, and exercise philosophy. Perception of change reflected participants’ feelings regarding changes in their bodies, emotions, and eating habits during the weight loss journey. This category included five subcategories: healthy eating, body feeling-negative, body feelings-positive, weight concerns, and emotional changes.

**Table 4:** Results of content analysis of participants’ daily weight loss summaries

Category (%)	Subcategory	Frequency	Definition	Example
Weight loss awareness (28.10%)	Risk situations	55	Risky situations where overeating behavior or excessive caloric intake is likely to occur.	“ Dinners are a stumbling block on the road to weight loss”; “You can’t be too hungry, it’s easy to bounce back”
	Self-motivation	54	Encourage themselves and stick to losing weight.	“Keep going, keep going!”; “For a healthier self. Come on!”
	Disadvantages	37	Factors that are not beneficial to weight loss	“Staying up late is truly detrimental to weight loss”; “It’s so hard to do exercise during menstruation (emoji: cry)”
	Keeping awareness	36	Being aware of one’s body	“Stay aware at all times”; “Ask myself if I’m hungry before I eat, and if I’m not, I don’t eat”
	Follow-up plan	29	Participants’ follow-up plans for their weight loss practice	“Continue to work hard on weight loss tomorrow… Go! Go! Go!“; “Eat less high-caloric food next week”
	Keeping patient	27	Keep more patience in the weight loss process, accept oneself, and stick to good habits.	“Losing weight is a constant battle.“; “Make it a routine”
Eating behavior (22.55%)	Restrictive diet	65	Participants reduce eating behavior and caloric intake	“I didn’t have dinner today”; “I only had red bean porridge this evening”
	Overeating	58	Participants exhibited overeating behavior or excessive caloric intake.	“I ate too much for dinner again… “; “Today’s calorie intake is bursting”
	Inhibit failure	38	Failed in the hope of not eating or eating less high-caloric foods	“I drank tea with milk today, ate high-caloric food, did not control my mouth”; “Always have various reasons to eat and drink”
	Irregular diet	13	Not eating at the proper time	“I didn’t eat dinner when I was busy at work”; “My diet is very irregular these two days”
	Mindful eating	12	Focusing on the eating process and experiencing the process of food entering the body	“Today I am continuing to try mindful eating”; “It feels good to concentrate on tasting food”
	Intuitive eating	5	Not limiting the amount of food intake and stopping when feeling full	“I can eat whenever I want, as long as I don’t eat too much”; “It feels good to be full”
Physical activity (16.88%)	Exercise amount	77	The participants’ exercise implementation.	“I worked late tonight and didn’t do any exercise”; “I went out today and did a lot of exercises”
	Type of exercise	48	Participants took different ways to exercise such as walking, aerobics, running, and swimming.	“Went out for a 9-kilometer walk after dinner”; “Insisted on walking for half an hour and jogging for half an hour “
	Exercise Philosophy	18	Perceptions of the relationship between exercise and weight loss.	“You can’t stop exercising; life is exercise”
Perception of change (32.47%)	Healthy eating	98	Participants eat healthier, pay attention to food calories, and have increased inhibition of high-caloric foods.	“I didn’t eat any snacks today, which is great, I’ve learned to control it”; “I feel like I’ve found the balance of diet adjustment”
	Body feeling-negative	84	Participants felt unwell, had a change in appetite, and did not sleep well.	“Very tired, and feel that my body cannot hold on”; “Last night did not sleep well again”
	Body feeling-positive	30	Participants felt good about the weight loss.	“I’m starting to get used to the rhythm now, and it feels good to sweat profusely”; “It feels good to have an empty stomach”
	Weight concerns	45	The participants are concerned about changes in their weight and experience weight loss or regain.	“This morning, I lost a little bit of weight again. Every day’s weight loss is a confirmation of my efforts”; “I’m sure I’m gaining weight, which is depressing”
	Emotional change	18	Participants experience negative emotions such as anxiety and depression and try to adjust.	“Just not in a good mood today”; “Have negative emotions but adjusted well”

Note. The percentage of a category is the ratio of the frequency of that category to the total number of meaning units included in the analysis (*N* = 847)

A chi-square test was conducted to reveal differences among the three groups (i.e., poor, moderate, and excellent) in different categories and subcategories. The results (Table 5) showed significant differences in frequency distribution across four categories and fifteen subcategories. Specifically, the poor group had lower observed frequencies than expected frequencies in all four categories. Additionally, the observed frequencies of the poor group were much lower than the expected frequencies in several subcategories, including “self-motivation”, “keeping awareness”, “follow-up plan”, “restrictive diet”, “overeating”, “irregular diet”, “mindful eating”, “exercise amount”, “type of exercise”, “body feeling-positive” and “weight concerns”. The frequency of daily log recorded by participants in the moderate group was also lower than the expected frequency in all four categories. Furthermore, their observed frequencies in the following subcategories: “risk situations”, “self-motivation”, “disadvantages”, “keeping awareness”, “keeping patient”, “restrictive diet”, “irregular diet”, “mindful eating”, “exercise amount”, “type of exercise”, and “healthy eating” were lower than the expected values. In contrast, the excellent group had higher observed frequencies than expected frequencies in all four categories and fifteen subcategories. These differences suggest that the group with better weight loss outcomes exhibits better self-monitoring adherence. They reported more frequencies in qualitative self-monitoring, had more self-awareness, recorded more eating behaviors, and physical activities, and perceived more changes triggered by weight loss. On the contrary, participants with less weight loss had less self-motivation and awareness, fewer records of restrictive diets and physical activities, and less tracking of weight.

**Table 5:** Results of chi-square test analysis of qualitative data for self-monitoring in each group

Category /Subcategory	Poor group		Moderate group		Excellent group		*X* ^2^
	observed	expected	observed	expected	observed	expected	
**Weight loss awareness**	61	74	49	86	128	78	49.916^***^
Risk situations	22	17	8	20	25	18	11.087^**^
Self-motivation	11	17	15	19	28	18	8.995^*^
Disadvantages	11	12	6	13	20	12	9.132^*^
Keeping awareness	6	11	7	13	23	12	15.759^***^
Follow-up plan	4	9	9	10	16	10	7.422^*^
Keeping patient	7	8	4	10	16	9	9.356^**^
**Eating behavior**	34	60	59	69	98	63	32.23^***^
Restrictive diet	12	20	16	23	37	21	17.215^***^
Overeating	8	18	25	21	25	19	8.297^*^
Inhibit failure	10	12	13	14	15	12	0.834
Irregular diet	1	4	2	5	10	4	11.538^**^
Mindful eating	3	4	1	4	8	4	6.887^*^
**Physical activity**	18	45	41	51	84	47	47.249^***^
Exercise amount	7	24	23	28	47	25	31.527^***^
Type of exercise	8	15	9	17	31	16	21.963^***^
Exercise philosophy	3	6	9	6	6	6	2.194
**Perception of change**	74	86	75	99	126	90	21.582^***^
Healthy eating	38	31	23	35	37	32	6.826^*^
Body feeling - negative	20	27	26	30	38	28	6.002
Body feeling - positive	2	9	10	11	18	10	12.590^**^
Weight concerns	8	14	7	16	30	15	23.522^***^
Emotional change	6	6	9	6	3	6	2.437

Note. The expected frequency was maintained as an integer. ^*^p<.05. ^**^p<.01. ^***^p<.001

## Discussion

In this study, we attempted to present a more comprehensive picture of participants’ self-monitoring behavior during a short-term online obesity intervention. We observed some fluctuation in participants’ self-monitoring data throughout the intervention. Furthermore, we found that some baseline characteristics of participants (e.g., weight loss motivation) and self-monitoring behaviors (e.g., timeliness of daily data filling) are positively correlated with their final weight loss outcomes. In addition., we categorized participants’ qualitative self-monitoring data into four distinct categories, and we observed differences in the distribution of these categories among groups with varying weight loss outcomes. These findings highlight the potential importance of self-monitoring behaviors to successful weight loss outcomes.

### The changes in participants’ self-monitoring

Participants’ self-monitoring data underwent interesting changes during the weight loss process. Throughout the intervention period, there was a gradual decrease in participants’ caloric intake, which aligns with findings from previous studies [33]. This implies that participants have learned to better control their caloric intake, which provides favorable conditions for achieving weight loss. It is worth noting that there was a slight increase in caloric intake during the last week than in week 4 though the difference was not statistically significant. Week 5 marked one week after the final intervention, and although participants were still expected to engage in self-monitoring, there seemed to be some subtle variations in their level of commitment. This increase raises concerns about the potential for a rebound in caloric intake.

In this study, participants’ daily sedentary hours gradually increased. This could be attributed to two potential reasons. Firstly, the intervention in this study might have placed more emphasis on healthy eating than physical activity. Secondly, the intervention occurred between June and September 2022, a time when China encountered an exceptionally scorching summer and people refrained from outdoor activities. While there may be some inconsistencies in research findings across different regions, previous studies have consistently demonstrated a close association between weather and sedentary behavior [34–36]. For instance, research on adults in the United Kingdom [34] and children in Denmark [37] found that participants tended to have longer sedentary periods during the winter. However, a survey of adolescents in Hong Kong, China, indicated an association between higher temperatures and increased sedentary time on weekends [36]. Therefore, in future studies of a similar nature, it is essential to consider the impact of weather factors on the effectiveness of weight loss interventions.

Furthermore, participants’ daily weight loss satisfaction showed a gradual decline over five weeks. The satisfaction levels were significantly higher in the first, second, and third weeks compared to the fifth week. Interestingly, this pattern mirrored the average weight loss, with participants achieving the greatest weight loss in the first week and the least in the last week. The difference between these two weeks was statistically significant. This observation suggests that participants were aware of their weight loss behavior and changes in their weight. As time progressed, there was a noticeable decrease in their satisfaction with their dietary restrictions or physical activity. This decline in satisfaction was consistent with the observed trend in their weight, strengthening the link between their efforts and the outcomes they achieved.

The frequency of daily logs also exhibited a gradual decrease over the five-week period. Since the daily log was optional for participants, this change may suggest a decrease in motivation to keep a summary or that participants had become accustomed to their weight loss behavior.

Taken together, these findings suggest that participants were more engaged and motivated during the initial stages of the intervention, but their adherence gradually declined, which is consistent with the findings of previous studies [38, 39]. Previous studies have demonstrated that early weight loss plays a crucial role in achieving eventual weight loss success [40, 41]. This indicates that practitioners may take advantage of participants’ initial high level of engagement and make efforts to enhance their engagement in the later stages.

### Predictors of weight loss

Identifying effective predictors of weight loss is clinically significant. Our results showed that baseline BMI, weight loss motivation, and timeliness of daily self-monitoring data filling could predict the final weight loss.

Higher baseline BMI among participants may indicate greater dissatisfaction with their weight [42] and a higher potential for weight loss, which in turn facilitated their weight loss progress. This finding is consistent with the results of Sasdelli et al. [43]. Additionally, participants’ weight loss motivation was found to significantly predict their final weight loss. The finding not only emphasizes the importance of motivation in weight loss interventions but also suggests that enhancing participants’ motivation can be a valuable approach in obesity interventions.

Moreover, the timeliness of participants’ daily data filling was found to be an effective predictor of weight loss. The act of daily data filling allowed participants to self-monitor their progress, which is beneficial for weight loss [44, 45]. Timely data filling could also be considered as adherence to the self-monitoring requirements, and previous studies have found that higher adherence is associated with greater weight loss [44, 46]. Interestingly, participants’ higher self-monitoring adherence contributed to weight loss that may not be moderated by participants’ gender. Comparable patterns have been observed in previous internet-based intervention studies that exclusively involved either males [47] or females [48], as well as both genders [44]. Despite the predominantly adult female participants in this study, the findings once again underscore the importance of adherence to online weight interventions.

In this study, no significant relationship was found between daily caloric intake, daily physical activity expenditure, and weight loss. This could be attributed to the data collection method used in this study. Due to the challenges of accurately weighing their daily food intake, there could have been potential errors in the participants’ estimates of food weight, which in turn affects the final caloric value. Moreover, prior evidence based on a large sample of wearable devices indicates that while physical activity contributes to modest weight reduction, achieving clinically significant weight loss remains a considerable challenge [49]. The findings of this study similarly reveal a non-significant association between physical activity expenditure and weight loss. As highlighted by Swift et al. [50], achieving clinically meaningful weight loss through aerobic exercise is challenging unless the training volume is exceptionally high. The greater value of physical activity and aerobic exercise in weight loss lies in enhancing physical function, particularly in reducing cardiovascular system risks [51].

Furthermore, emotional distress has long been recognized as a contributing factor to overweight or obesity [52], and some studies have incorporated emotional management as part of weight interventions [33, 53]. However, in this study, no significant association was found between participants’ daily mood and weight loss. This result may be attributed to the inclusion of the emotional intervention component in this study. Participants may have developed better emotional awareness and coping strategies, enabling them to identify their emotions and make adjustments, thus reducing the likelihood of engaging in emotional eating.

### Participants’ daily log

Our qualitative analysis of participants’ daily logs revealed four categories: *weight loss awareness*, *eating behavior*, *physical activity*, and *perception of change*. Among these categories, the *perception of change* was the most frequently mentioned, followed by *weight loss awareness*, *eating behavior*, and *physical activity*.

*Perception of change* encompasses participants’ perceptions of various aspects including their healthy eating behavior, body, weight, and emotion. The subcategory of healthy eating garnered the highest proportion of this category. This indicates that participants placed significant emphasis on their dietary choices and recognized the importance of adopting healthy eating habits during their weight loss journey. Negative body feelings were the second most frequently reported subcategory, this may be the result of changes in exercise and eating behavior during the weight loss process. In the beginning phase, the increase in physical activity can often result in muscle soreness and fatigue [54]. Also, the reduced intake of high-caloric foods may impact participants’ eating experience and hunger levels. Furthermore, participants also mentioned weight concerns, healthy eating, and emotions, indicating their growing awareness of both the physical and psychological changes during the weight loss process.

*Weight loss awareness* involved participants being mindful of risky situations, recognizing adverse factors in the weight loss process, motivating themselves, and maintaining patience. Previous studies have highlighted the importance of identifying and addressing high-risk situations as a strategy for long-term weight loss success [55], and our research findings support this view. Interestingly, some participants who encountered such risks quickly adjusted themselves and proposed follow-up weight loss plans.

A study on lifestyle interventions has shown that individuals who achieve successful weight loss exhibit increased control over their dietary behaviors [56]. In the qualitative self-monitoring data of this study, participants similarly recorded different eating behaviors. The *eating behavior* category included descriptions of both risky eating behaviors (e.g., overeating) and advocated eating behaviors (e.g., mindful eating). This demonstrates the participants’ efforts to modify their dietary habits during the weight loss process, which is crucial for successful weight reduction.

The category of *physical activity* records appeared less frequently in participants’ daily logs, which included descriptions of exercise amounts, types of exercise, and perceptions of exercise. The low rates in this category seem to be consistent with quantitative data (e.g., physical activity expenditure, and sedentary hours). This may be attributed to the hot weather during the intervention period, which could have hindered participants’ engagement in physical activities.

### Self-monitoring differences among participants with different weight loss outcomes

In the current study, the probability distribution of daily log frequencies reported by participants with different weight loss outcomes showed inconsistencies. Compared to the excellent group, both the poor group and moderate group reported lower observed frequencies than expected frequencies in all four categories and most subcategories. Participants in the excellent group exhibited higher frequencies in identifying risk situations, disadvantages, self-awareness, self-motivation, and follow-up weight loss plans, as well as demonstrating greater patience. Additionally, they reported more adjustments in eating habits, such as self-restriction and healthy eating, and recorded a greater frequency of physical activity, including different exercise types and levels, as well as expressing more weight concerns. Conversely, the poor group and moderate group demonstrated opposite characteristics overall. Considering that summarizing daily weight loss progress is an optional feature, participants’ willingness to fill out these logs implies a potentially higher level of self-monitoring adherence. Previous quantitative studies have confirmed that participants with high self-monitoring adherence experience more significant weight loss outcomes [16, 38]. In line with these findings, the present study also observed that the group exhibiting greater weight loss reported more daily logs, further supporting the value of self-monitoring adherence for weight loss.

It is noteworthy that there were also inherent inconsistencies in self-monitoring among the three groups. For example, the poor group reported higher than expected frequencies in “risk situations” and “healthy eating”. However, the moderate and excellent groups reported higher than expected frequencies of “overeating”. This may suggest that weight loss is a complex process. Participants who achieve less weight loss still demonstrate some positive weight loss behaviors, indicating their efforts in the process. On the other hand, participants who achieve more weight loss may also exhibit some unfavorable behaviors during their weight loss journey, highlighting the potential challenges and complexities involved in successful weight loss.

Furthermore, an intriguing observation is that, among the three participant groups, the excellent group reported a higher quantity of physical activity. However, quantitative self-monitoring data indicated that energy expenditure from physical activity and sedentary behavior could not predict the final weight loss. This seemingly contradictory phenomenon may be attributed to the fact that the reported physical activity in qualitative self-monitoring merely records behavior and may not accurately reflect the actual energy expenditure. The excellent group reported a greater frequency of recording physical activity, serving as an indication of high adherence to self-monitoring. Higher levels of self-monitoring contribute to sustained improvement in health behaviors [38], leading to more substantial weight loss.

### Strengths and limitations

This study has some notable strengths. Firstly, it provides valuable insights into how participants’ self-monitoring behavior changed during a short-term online group psychological weight loss intervention. Dieters demonstrated some promising (e.g., decreases in caloric intake) and worrying changes (e.g., increases in sedentary hours and gradual slowing of weight loss). These observations suggest a higher level of adherence in the early stages of weight loss and a potential risk of reduced motivation in the later stages. These patterns can be utilized by weight loss practitioners to develop more focused intervention strategies, such as targeting weight loss motivation and promoting timely self-monitoring. Additionally, providing appropriate support throughout the weight loss journey, such as group interventions and mutual monitoring, can further enhance the effectiveness of these interventions.

Secondly, the study took a comprehensive approach by analyzing participants’ self-monitoring data using a mixed method. The convergence of quantitative and qualitative data enhances the validity of the findings. For example, the quantitative data revealed a decrease in daily physical activity expenditure and an increase in daily sedentary activity, which was complemented by the qualitative data indicating a decrease in the frequency of physical activity categories. This combination of data provides unique and valuable insights into the intricacies of the weight loss process.

Thirdly, this study contributes to the existing knowledge of predictors of weight loss in online intervention studies. In addition to baseline BMI and weight loss motivation, participants’ timely filling in of the day’s self-monitoring data is also a critical contributing factor to weight loss. This finding underscores the importance of consistent and regular tracking behaviors.

Finally, this study examined differences in participants’ qualitative data. The results highlight that successful weight losers are more proactive and have more textual summaries of their daily weight loss performance.

However, there are some limitations to this study. Firstly, participants’ caloric intake relied on estimates obtained through a smartphone application. Participants may have inaccurately estimated food weights, leading to potential errors in the calculation of caloric intake. Future studies could consider incorporating more objective measurements (e.g., electronic scales and fitness trackers), to ensure more precise results. Secondly, caution should be exercised when generalizing the findings of this research. Due to the limited sample size of this study and its focus on Chinese adult females, it is important to exercise caution when generalizing the conclusions and considering potential differences in dietary structures and obesity perceptions.

## Conclusion

This study uncovered a fluctuating pattern in the self-monitoring behavior of participants undergoing an online group psychological weight loss intervention. The analysis of quantitative and qualitative data revealed that participants exhibited higher levels of engagement in self-monitoring during the initial stages of weight loss, which gradually declined in later stages. Notably, participants with higher baseline BMI, higher levels of weight loss motivation, and consistently engaged in timely self-monitoring achieved more significant weight loss outcomes. Furthermore, more successful participants reported more frequent and detailed content in their weight loss summary texts.

Based on these findings, clinical practitioners should strive to enhance both participants’ weight loss motivation and their adherence to self-monitoring. Incorporating digital technologies such as wearable devices and mobile apps, along with mutual reminders from group members, could prove beneficial in boosting self-monitoring adherence. This, in turn, facilitates heightened awareness of weight loss-related physical activity and dietary habits. Additionally, future researchers could further expand participant sample sizes and employ more precise measurement tools to assess daily calorie intake and expenditure, thereby enhancing the internal validity of the study.

## Data Availability

Following the confidentiality clause of informed consent, data from this study are not publicly available. De-identified data may be available upon specific request by contacting the corresponding author.

## References

[1] World Health Organization. Obesity and overweight. Fact sheets. 2021. https://www.who.int/news-room/fact-sheets/detail/obesity-and-overweight. Accessed 2 Dec 2022.

[2] Pan X-F, Wang L, Pan A (2021). Epidemiology and determinants of obesity in China. Lancet Diabetes Endocrinol.

[3] Chooi YC, Ding C, Magkos F (2019). The epidemiology of obesity. Metabolism.

[4] Powell-Wiley TM, Poirier P, Burke LE, Despres J-P, Gordon-Larsen P, Lavie CJ (2021). Obesity and Cardiovascular Disease: A Scientific Statement from the American Heart Association. Circulation.

[5] Halfon N, Larson K, Slusser W (2013). Associations between Obesity and Comorbid Mental Health, Developmental, and Physical Health conditions in a nationally Representative Sample of US children aged 10 to 17. Acad Pediatr.

[6] Yang J, Hu J, Zhu C (2021). Obesity aggravates COVID-19: a systematic review and meta-analysis. J Med Virol.

[7] Burke LE, Swigart V, Warziski Turk M, Derro N, Ewing LJ (2009). Experiences of Self-Monitoring: successes and struggles during treatment for weight loss. Qual Health Res.

[8] Hartmann-Boyce J, Boylan A-M, Jebb SA, Aveyard P (2019). Experiences of Self-Monitoring in Self-Directed weight loss and weight loss maintenance: systematic review of qualitative studies. Qual Health Res.

[9] Burke LE, Wang J, Sevick MA (2011). Self-monitoring in weight loss: a systematic review of the literature. J Am Diet Assoc.

[10] Rafiei N, Gill T (2018). Identification of factors contributing to successful self-directed weight loss: a qualitative study. J Hum Nutr Diet.

[11] Dunn CG, Turner-McGrievy GM, Wilcox S, Hutto B (2019). Dietary self-monitoring through calorie tracking but not through a Digital Photography App is Associated with Significant Weight loss: the 2SMART Pilot Study—A 6-Month Randomized Trial. J Acad Nutr Diet.

[12] Patel ML, Brooks TL, Bennett GG (2020). Consistent self-monitoring in a commercial app-based intervention for weight loss: results from a randomized trial. J Behav Med.

[13] Burke LE, Conroy MB, Sereika SM, Elci OU, Styn MA, Acharya SD (2011). The Effect of Electronic Self-Monitoring on Weight loss and Dietary Intake: a randomized behavioral weight loss trial. Obesity.

[14] Helsel DL, Jakicic JM, Otto AD (2007). Comparison of techniques for Self-Monitoring Eating and Exercise behaviors on Weight loss in a correspondence-based intervention. J Am Diet Assoc.

[15] Patel ML, Hopkins CM, Brooks TL, Bennett GG (2019). Comparing self-monitoring strategies for weight loss in a Smartphone App: Randomized Controlled Trial. JMIR MHealth UHealth.

[16] Harvey J, Krukowski R, Priest J, West D (2019). Log often, lose more: electronic dietary self-monitoring for weight loss. Obesity.

[17] Thomas JG, Bond DS, Phelan S, Hill JO, Wing RR (2014). Weight-loss maintenance for 10 years in the National Weight Control Registry. Am J Prev Med.

[18] Jensen CD, Duraccio KM, Hunsaker SL, Rancourt D, Kuhl ES, Jelalian E (2014). A qualitative study of successful adolescent and young Adult Weight losers: implications for Weight Control intervention. Child Obes.

[19] Hoffmann DA, Carels RA (2016). Does when you eat and exercise matter? Differences in eating and physical activity patterns in overweight and obese adults. Eat Weight Disord-Stud Anorex Bulim Obes.

[20] Mattila E, Lappalainen R, Parkka J, Salminen J, Korhonen I (2010). Use of a mobile phone diary for observing weight management and related behaviours. J Telemed Telecare.

[21] Phelan S, Wing RR, Raynor HA, Dibello J, Nedeau K, Peng W (2008). Holiday weight management by successful weight losers and normal weight individuals. J Consult Clin Psychol.

[22] Chang LU, Zhenghe W, Yanhui D, Jun MA (2017). Prevalence of weight-loss behaviors and the associations between weight-loss behaviors and weight-related perceptions among students in primary and middle schools in China. Chin J Sch Health.

[23] Turner-McGrievy GM, Tate DF (2013). Weight loss social support in 140 characters or less: use of an online social network in a remotely delivered weight loss intervention. Transl Behav Med.

[24] Volz K, Wyckoff E, Medina TH, Denmat Z, Field C, LaRose J (2021). Impact of income and perceived stress on engagement and weight loss outcomes in an online behavioral weight loss program. J Behav Med.

[25] Verster JC, Mulder KEw V, Van MCe EC, Hendriksen PA, Scholey A (2023). Test-retest reliability of single-item assessments of immune fitness, mood, and quality of life. Heliyon.

[26] Varfi N, Rothen S, Jasiowka K, Lepers T, Bianchi-Demicheli F, Khazaal Y (2019). Sexual Desire, Mood, attachment style, Impulsivity, and self-esteem as predictive factors for addictive cybersex. JMIR Ment Health.

[27] Lindgren B-M, Sundbaum J, Eriksson M, Graneheim UH (2014). Looking at the world through a frosted window: experiences of loneliness among persons with mental ill-health: looking at the world through a frosted window. J Psychiatr Ment Health Nurs.

[28] Qi J (2023). Fprt. Chinese College Students’ death cognition and its long-term changes after Wenchuan Earthquake. OMEGA - J Death Dying.

[29] Goldstein SP, Thomas JG, Brick LA, Zhang F, Forman EM (2021). Identifying behavioral types of dietary lapse from a mobile weight loss program: preliminary investigation from a secondary data analysis. Appetite.

[30] Thomas JG, Raynor HA, Bond DS, Luke AK, Cardoso CC, Foster GD (2017). Weight loss in Weight watchers Online with and without an activity tracking device compared to control: a randomized trial: Weight watchers Online. Obesity.

[31] Toon J, Geneva M, Sharpe P, Lavin J, Bennett S, Avery A (2022). Weight loss outcomes achieved by adults accessing an online programme offered as part of Public Health England’s ‘Better Health’ campaign. BMC Public Health.

[32] Miles J. Tolerance and variance inflation factor. Wiley StatsRef: statistics Reference Online. John Wiley & Sons, Ltd; 2014.

[33] Simon GE, Rohde P, Ludman EJ, Jeffery RW, Linde JA, Operskalski BH (2010). Association between change in depression and change in weight among women enrolled in weight loss treatment. Gen Hosp Psychiatry.

[34] O’Connell SE, Griffiths PL, Clemes SA (2014). Seasonal variation in physical activity, sedentary behaviour and sleep in a sample of UK adults. Ann Hum Biol.

[35] Ferguson T, Curtis R, Fraysse F, Lagiseti R, Northcott C, Virgara R (2021). Annual, seasonal, cultural and vacation patterns in sleep, sedentary behaviour and physical activity: a systematic review and meta-analysis. BMC Public Health.

[36] Zheng C, Huang WY, Wong SH-S (2019). Associations of weather conditions with adolescents’ daily physical activity, sedentary time, and sleep duration. Appl Physiol Nutr Metab.

[37] Hjorth MF, Chaput J-P, Michaelsen K, Astrup A, Tetens I, Sjödin A (2013). Seasonal variation in objectively measured physical activity, sedentary time, cardio-respiratory fitness and sleep duration among 8–11 year-old Danish children: a repeated-measures study. BMC Public Health.

[38] Butryn ML, Godfrey KM, Martinelli MK, Roberts SR, Forman EM, Zhang F (2020). Digital self-monitoring: does adherence or association with outcomes differ by self‐monitoring target?. Obes Sci Pract.

[39] Turner-McGrievy GM, Dunn CG, Wilcox S, Boutté AK, Hutto B, Hoover A (2019). Defining adherence to Mobile Dietary Self-Monitoring and assessing Tracking over Time: tracking at least two eating occasions per day is best marker of adherence within two different Mobile Health Randomized Weight loss interventions. J Acad Nutr Diet.

[40] Barnes RD, Ivezaj V, Pittman BP, Grilo CM (2018). Early weight loss predicts weight loss treatment response regardless of binge-eating disorder status and pretreatment weight change. Int J Eat Disord.

[41] Patel ML, Hopkins CM, Bennett GG (2019). Early weight loss in a standalone mHealth intervention predicting treatment success. Obes Sci Pract.

[42] Pappa GL, Cunha TO, Bicalho PV, Ribeiro A, Couto Silva AP, Meira W (2017). Factors Associated with Weight Change in Online Weight Management communities: a Case Study in the LoseIt Reddit Community. J Med Internet Res.

[43] Sasdelli AS, Petroni ML, Delli Paoli A, Collini G, Calugi S, Dalle Grave R (2018). Expected benefits and motivation to weight loss in relation to treatment outcomes in group-based cognitive-behavior therapy of obesity. Eat Weight Disord - Stud Anorex Bulim Obes.

[44] Jacobs S, Radnitz C, Hildebrandt T (2017). Adherence as a predictor of weight loss in a commonly used smartphone application. Obes Res Clin Pract.

[45] Yan L (2020). The kindness of commenters: an empirical study of the effectiveness of Perceived and received support for weight-loss outcomes. Prod Oper Manag.

[46] Tanaka K, Sasai H, Wakaba K, Murakami S, Ueda M, Yamagata F (2018). Professional dietary coaching within a group chat using a smartphone application for weight loss: a randomized controlled trial. J Multidiscip Healthc.

[47] Behr H, Ho AS, Yang Q, Mitchell ES, DeLuca L, Greenstein N (2023). Men’s weight loss outcomes, behaviors, and perceptions in a Self-Directed Commercial Mobile Program: retrospective analysis. Health Educ Behav.

[48] Webber KH, Tate DF, Ward DS, Bowling JM (2010). Motivation and its relationship to adherence to self-monitoring and weight loss in a 16-week internet behavioral weight loss intervention. J Nutr Educ Behav.

[49] El Fatouhi D, Delrieu L, Goetzinger C, Malisoux L, Affret A, Campo D (2021). Associations of Physical Activity Level and Variability with 6-Month Weight Change among 26,935 users of connected devices: Observational Real-Life Study. Jmir Mhealth Uhealth.

[50] Swift DL, Johannsen NM, Lavie CJ, Earnest CP, Church TS (2014). The role of Exercise and physical activity in weight loss and maintenance. Prog Cardiovasc Dis.

[51] Swift DL, McGee JE, Earnest CP, Carlisle E, Nygard M, Johannsen NM (2018). The effects of Exercise and physical activity on weight loss and maintenance. Prog Cardiovasc Dis.

[52] Puder JJ, Munsch S (2010). Psychological correlates of childhood obesity. Int J Obes.

[53] Stapleton P, Lilley-Hale E, Mackintosh G, Sparenburg E (2020). Online delivery of Emotional Freedom techniques for Food cravings and Weight Management: 2-Year Follow-Up. J Altern Complement Med.

[54] Zdziarski LA, Wasser JG, Vincent HK (2015). Chronic pain management in the obese patient: a focused review of key challenges and potential exercise solutions. J PAIN Res.

[55] Greaves C, Poltawski L, Garside R, Briscoe S (2017). Understanding the challenge of weight loss maintenance: a systematic review and synthesis of qualitative research on weight loss maintenance. Health Psychol Rev.

[56] Nurkkala M, Kaikkonen K, Vanhala ML, Karhunen L, Keränen A-M, Korpelainen R (2015). Lifestyle intervention has a beneficial effect on eating behavior and long-term weight loss in obese adults. Eat Behav.

